# Air pollution in Iran: The current status and potential solutions

**DOI:** 10.1007/s10661-023-11296-5

**Published:** 2023-05-26

**Authors:** Farzaneh Taghizadeh, Babak Mokhtarani, Nejat Rahmanian

**Affiliations:** 1 Chemistry and Chemical Engineering Research Center of Iran, P. O. Box 14335-186, Tehran, Iran; 2 Department of Chemical Engineering, Faculty of Engineering and Informatics, University of Bradford, Bradford, BD7 1DP UK

**Keywords:** Air pollution, Particulate matter, Mitigation strategy, Air quality management, Iran

## Abstract

Air pollution has been integrated into global challenges over the last few years due to its negative impact on the health of human beings, increasing socio-economic risks, and its contribution to climate change. This study attempts to evaluate the current status of Iran’s air pollution with regard to the sources of emissions, control policies, and the health and climate consequences that have resulted through available data from monitoring stations reported in the literature, official documents, and previously published papers. Many large cities in Iran surpass the permissible concentration of air pollutants, particularly particulate matter, sulfur dioxide, black carbon, and ozone. Although regulations and policies are in place and enormous efforts are being made to address air pollution issues in the country, implementation and enforcement are not as effective as they could be. The significant challenges may be regarded as the inefficiency of regulation and supervision systems, the lack of air quality monitoring systems and technology, particularly in industrial cities rather than Tehran, and the lack of continual feedback and investigations on the efficiency of regulation. Providing such an up-to-date report can bring opportunities for international collaboration, which is essential in addressing air pollution worldwide. We suggest that a way forward could be more focused on conducting systematic reviews using scientometric methods to show an accurate picture and trend in air pollution and its association in Iran, implementing an integrated approach for both climate change and air pollution issues, collaborating with international counterparts to share knowledge, tools, and techniques.

## Introduction

Industrialization, particularly among developing nations, has been a key factor contributing to environmental degradation. Air pollution, caused by industrialization, is one of the ten most significant environmental health issues linked to increasing mortality rates worldwide. (Hajizadeh et al., 2020; Singh et al., 2022). According to research, this is the result of a number of factors, such as the increased number of private vehicles, unmaintained vehicles, low-quality fuel, poorly paved roads, and inadequate infrastructure in urban areas. As a result, air pollution control is complex and requires strict environmental regulations (Adami et al., 2022; Kumar et al., 2023). Although the adverse effects of air pollutants are particularly evident within the boundaries of cities, this pollution is not restricted to the urban centers’ boundaries but can be transported over extensive distances and contribute to the general hemispheric background pollution (Gurjar et al., 2016; Molina, 2021). On this account, air pollution should be considered a long-term global change issue rather than a local concern (Baklanov et al., 2016).

According to the Environmental Protection Agency (EPA), in fuel combustion, pollutants are classified as either criteria or non-criteria pollutants. Generally, six major air pollutants are considered to be responsible for air pollution (also known as “criteria air pollutants”), namely carbon monoxide (CO), particulate matter (PM10) (PM2.5), ozone (O_3_), sulfur dioxide (SO_2_), and nitrogen dioxide (NO_2_) (Dastoorpoor et al., 2019). GHG emissions from cities and industrial locations mainly consist of carbon dioxide (CO_2_), methane (CH_4_), ozone (O_3_), and halocarbons. Nevertheless, the polluted atmospheres of metropolitan areas contain large amounts of particulate matter (PM), which is the result of human activity and natural sources. Sulfur dioxide and nitrogen oxides are also transformed into potent acids by photochemical reactions regionally and continentally, resulting in acid rain (Baklanov et al., 2016; Molina, 2021; Ramanathan, 2020). Some characteristics of criteria air pollutants and the resulting impacts on health are listed in Table 1.

**Table 1:** Characteristics of criteria air pollutants (US EPA website)

Pollutant	Sources	Environmental and human health risks
PM	Diesel engines, power plants, industries, dust blown by the wind	Irritation of the eyes, lung disease, cancer, cardiovascular disease, asthma, bronchitis, impaired visibility, negative esthetic effects
Carbon monoxide (CO)	Vehicle exhaust, fires, industrial processes, and indoor sources	Chronic headaches, impaired mental alertness, heart disease, fetal development problems, and death
Nitrogen oxide (NO_2_)	Vehicle exhaust, power plants, and other domestic and industrial sources that use fossil fuels	Respiratory infections and lung irritation. Result in smog formation, acid deposition, degradation of water quality, climate change, and impaired visibility
Sulfur dioxide (SO_2_)	Energy production, burning fossil fuels, manufacturing processes, exhaust emissions from vehicles	Eye irritation, coughing, breathing difficulties damage to the respiratory system
Lead (Pb)	Glass and cement manufacturers, metal processing, incineration of waste	Hypertension, kidney and brain disease, cancer. Affects aquatic ecosystems, animals, and the climate
Ozone (O_3_)	Resulting from the reaction between sunlight and other pollution as well as vehicle exhaust	Ecosystem destruction, climate change. Irritation of the eyes and throat, problems with the respiratory system

**Fig. 1:**
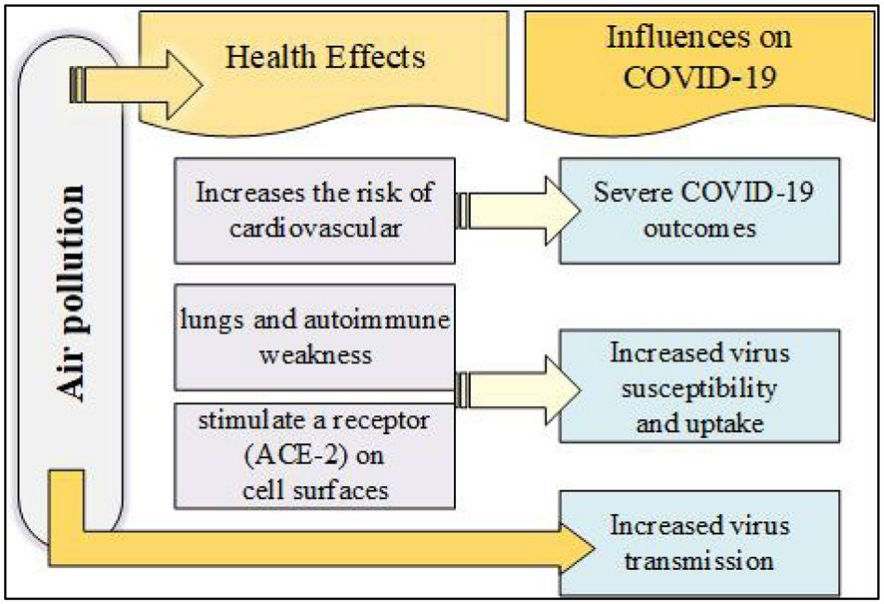
Air pollution and COVID-19 association

The rapid decline in global air quality is having a detrimental effect on human health, the economy, and ecosystems (Anjum et al., 2021). Long-term exposure to air pollution has been linked to a decrease in life expectancy, as well as an increase in cardiovascular and respiratory diseases. Even at low exposure levels, there are significant impacts on public health (A Pozzer et al., 2023). Moreover, the risk of mortality from COVID-19 can be attributed to air pollution. Recent related studies illustrated that the population exposed to a higher level of air pollution during their lifetime was more likely to have adverse respiratory results. This provides further support for integrating air pollution reduction strategies with control measures against COVID-19 transmission. It is estimated that between 7 and 33% of COVID-19 deaths are related to sustained exposure to polluted air (IQAir, 2021; Kurwadkar et al., 2023). Various factors related to air pollution and increased vulnerability to COVID-19, according to recent research, are illustrated in Fig. 1. Furthermore, most countries have implemented national lockdown and social distancing policies resulting in reduced industrial, commercial, and human activity, followed by reduced air pollution emissions and improved air quality. Several studies conducted around the world support this argument that air pollutants’ reduction in the atmosphere has been observed as a result of national lockdowns confirming that national lockdowns led to air pollutants’ reduction in the atmosphere (Gautam & Gollakota, 2023; Kiyan et al., 2023; Andrea Pozzer et al., 2020; Rodríguez-Urrego, 2020).

Climate change and air pollution are aggravated by the combustion of fuel, which increases CO_2_ emissions that cause global warming (Taghizadeh et al., 2020). Simultaneously, the generation of other pollutants, such as NO_x_, SO_2_, and PM, is the main cause of air pollution (Baklanov et al., 2016; Parrish et al., 2016; Ramanathan, 2020).

Considering the above, this report provides an updated assessment of the current deteriorated air quality status in Iran, a country located in Western Asia, which has its own share of problems; as a result, air quality is adversely affected. Providing such comprehensive studies not only can help authorities to plan and adopt effective strategies but also may provide a vivid picture of Iran’s atmospheric environmental situation and its contribution to the global climate. Many cities in Iran have well-established monitoring stations for monitoring air quality. Air pollution has been the subject of a large number of scientific reports, a number that is growing rapidly. The time is ripe for a review of the current state of knowledge regarding air pollution to identify issues related to understanding and controlling air pollution in this country. In this regard, we first review the current air quality status and system in Iran through available documents and publications. Then, a general overview of the research published has been discussed. Finally, we suggest several practical suggestions that can be used for the next step of this issue.

## The current air quality of Iran

### Fundamental information about Iran

**Fig. 2:**
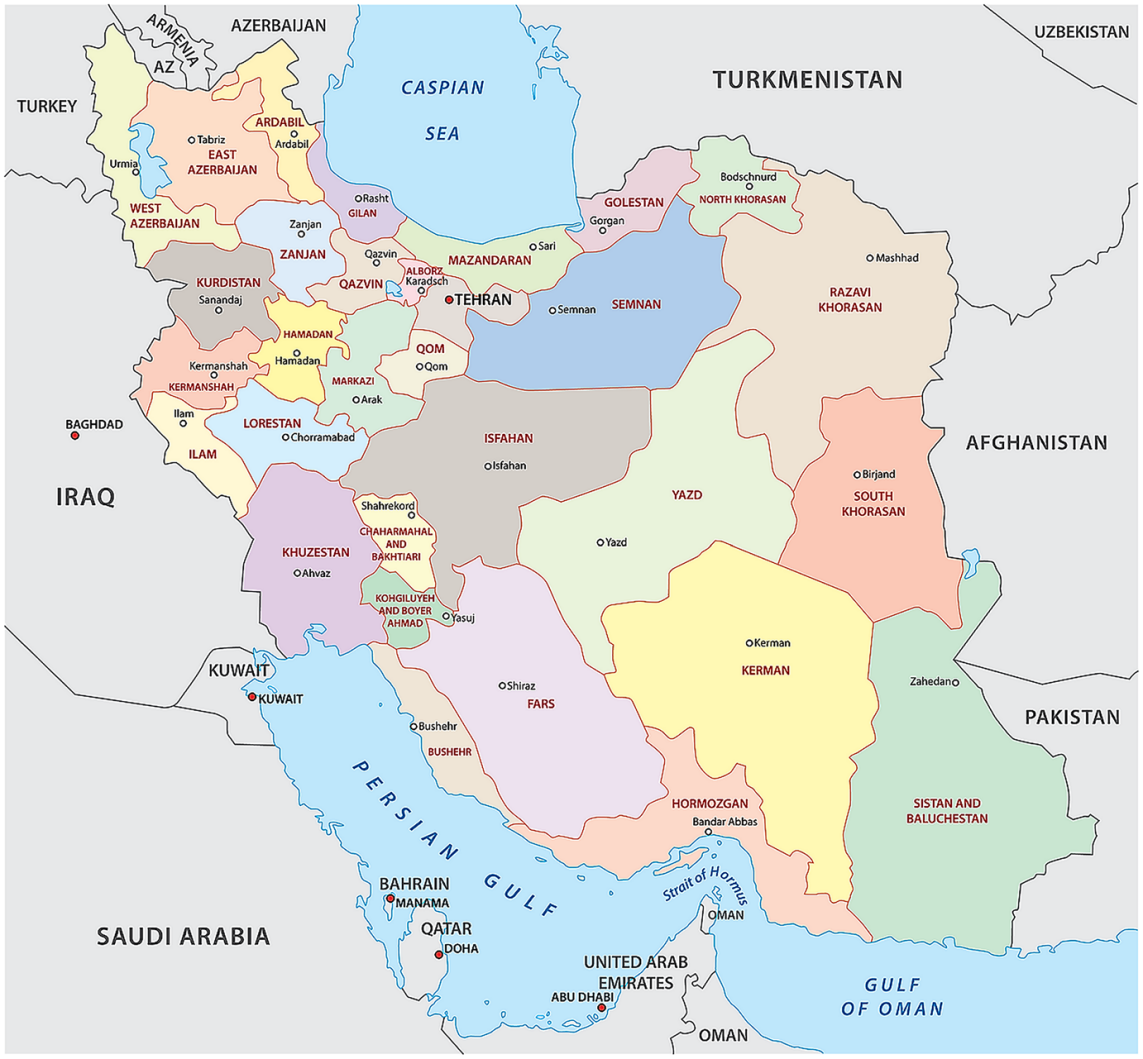
Provinces of Iran map from World Atlas (https://www.worldatlas.com/maps/iran)

Iran is divided into 31 provinces (see overall map in Fig. 2) bordering other countries in the west such as Iraq, and Turkey, and in the eastern Afghanistan and Pakistan. The capital and largest city of Iran, Tehran, is an influential political, economic, and cultural center, with over 8.8 million residents. Iran has a population of 83.2 million according to the last census held in 2018. Iran has two separate organizations that monitor ambient air pollutants on a regular basis: (1) the Department of Environment (DoE), headed by a deputy vice president, and (2) the Air Quality Control Company (AQCC), which only monitors ambient air pollutants in some cities. The measurements in both of them are based on beta-radiation attenuation monitors or beta-gauge instruments (model MP 101 M of Environment SA, France; FH 62 IN, FAG Kugelfischer, Germany; APDA-351E of Horiba, Japan; and instruments of Ecotech, Australia) (Shamsipour et al., 2019). Currently, there are thirty-nine stations in Tehran and around 200 nationwide. However, it is not clear how many stations are working. This situation may be attributed to the limited funds of the Environmental Protection Agency as well as the high cost of maintaining and operating stations.

**Fig. 3:**
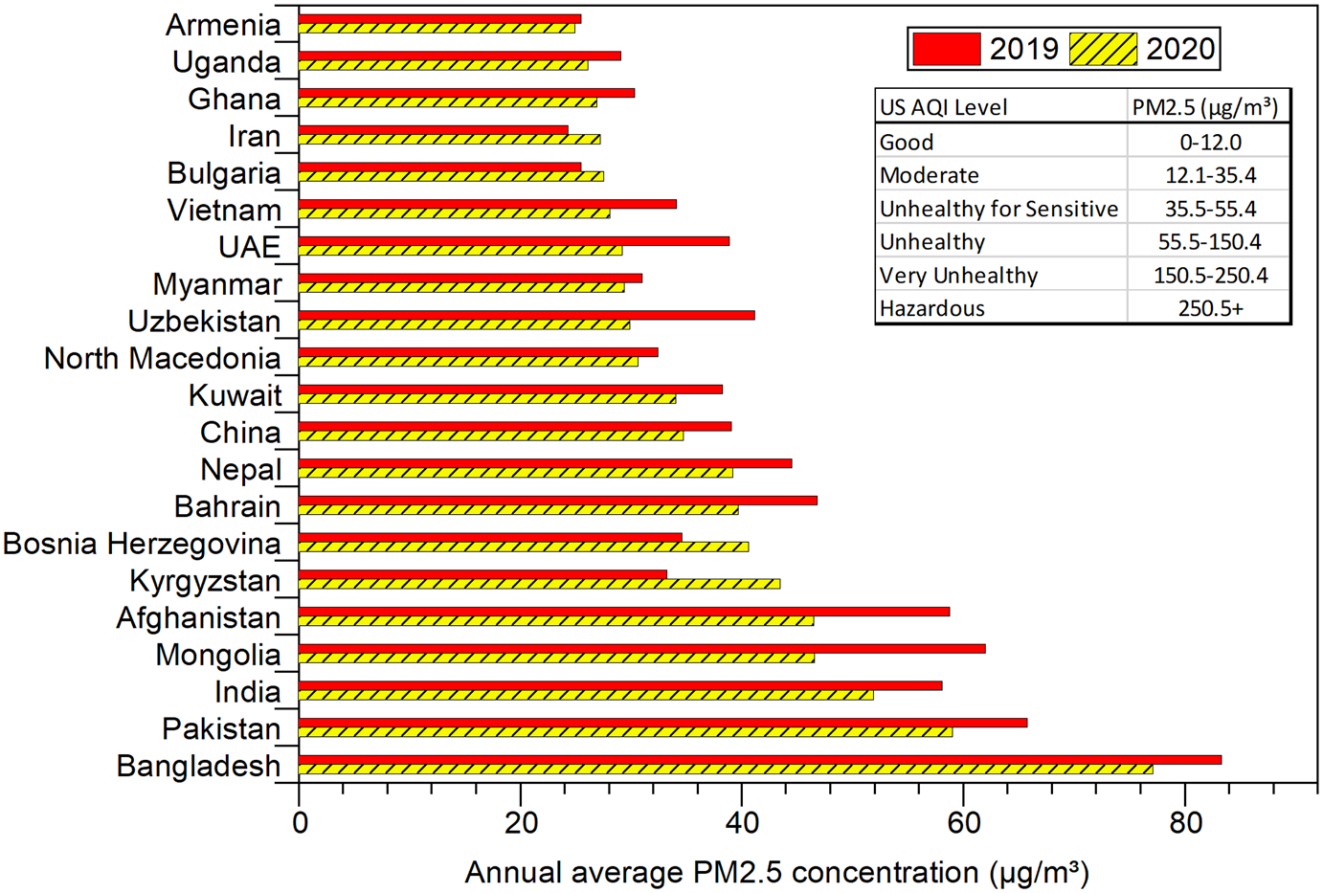
The annual average concentration of PM2.5 (μg/m.^3^), based on population in 2020 and 2019, table inside the figure: US Air Quality Index (AQI) for PM2.5 (adapted from world air quality report (IQAir, 2020, 2019))

**Fig. 4:**
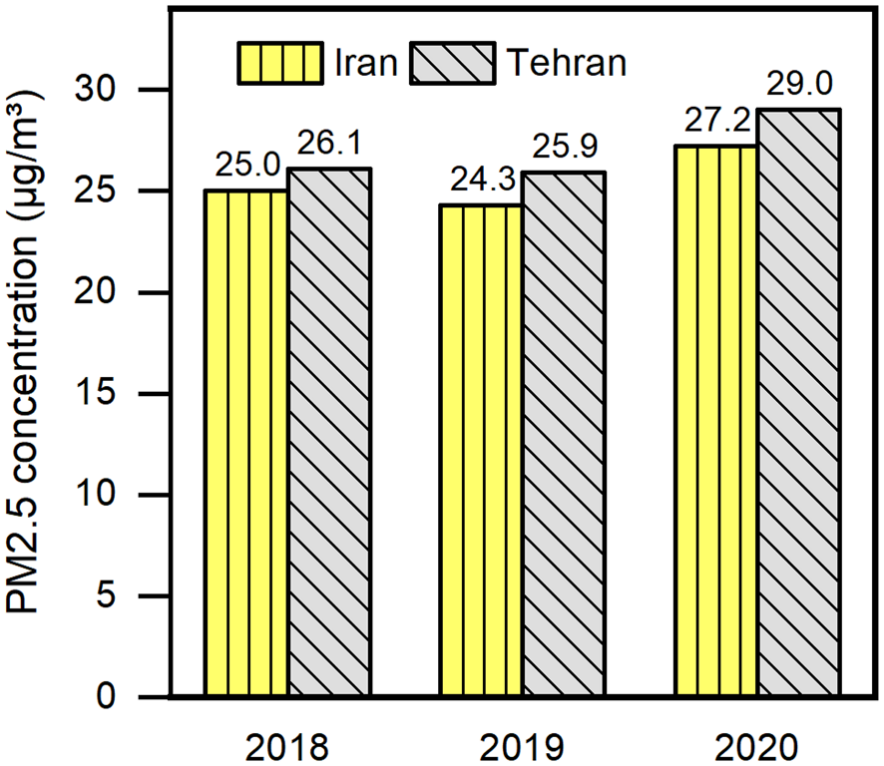
Annual average PM2.5 concentration in Iran and Tehran (adapted from world air quality report (IQAir, 2020, 2019))

Regarding the 2020 world air quality report by IQAir (2020), based on average PM2.5 concentration (g/m^3^) depending on population, Iran records 27.2 μg/m^3^ which is more than two times above the WHO exposure recommendation; this places it in a “moderate” status and 23rd place in the ranking of all countries. The average annual level of PM2.5 (μg/m^3^), for several polluted world counties, is illustrated in Fig. 3. As can be seen in Fig. 3 in 2020, although many countries observed reductions in PM2.5, Kyrgyzstan (+ 31.0%), Bosnia Herzegovina (+ 17.3%), Iran (+ 11.9%), and Bulgaria (+ 7.8%) experienced increases as compared with 2019. Besides, Tehran, the capital city of Iran, with 29.0 μg/m^3^, ranked 19th place in the world capital city ranking. Moreover, according to data annually reported by IQAir, the annual average PM2.5 concentration in 2018, 2019, and 2020 in Iran and its capital (Tehran) is presented in Fig. 4 (IQAir, 2021; Andrea Pozzer et al., 2020; WHO, 2020).

There are many major cities worldwide where PM2.5 levels decrease annually as well as observed visibly cleaner air in 2020 compared to 2019 as a result of COVID-19-related lockdowns (Chossière et al., 2021). However, PM2.5 levels in Iran increased from 24.3 to 27.2 μg/m^3^; that is a 10.66% increment. In the case of Tehran, this was raised to about 10.68%. According to the recent report published by the Iran Air Quality Company (Air Quality Control Company 2021), this might be due to more preference to use of the private car rather than public transportation during the period of COVID-19 lockdowns. The average monthly Air Quality Index based on the concentration of PM2.5 for 38 regions in Iran in 2020 as well as the annual average of recent years (2017–2020) is summarized in Table 2.

**Table 2:** Average Air Quality Index with the legend of PM2.5 for 38 regions in Iran

	Region	2020 AVG	JAN	FEB	MAR	APR	MAY	JUN	JUL	AUG	SEP	OCT	NOV	DEC	2019 AVG	2018 AVG
1	Ahvaz	39.5	37.2	38.5	32.6	25.9	40.3	29.8	47.9	30.2	50.5	52.0	47.3	39.1	-	-
2	Qarchak	39.5	47.6	38.7	30.8	24.9	27.4	35.5	-	29.8	35.1	49.2	37.4	59.9	-	-
3	Pishva	37.9	39.0	33.8	26.9	22.9	32.9	36.4	39.4	48.7	61.2	49.5	30.9	49.4	-	-
4	Mahshahr	37.2	31.7	34.2	31.1	24.7	40.2	33.1	47.7	32.9	43.8	44.1	40.7	37.0	-	-
5	Andimeshk	34.4	32.9	28.8	24.7	28.9	40.6	33	44.4	38.7	45.0	36.4	33.9	26.1	32.1	39.2
6	Shooshtar	33.9	33.7	28.3	24.6	24.9	41.8	35.4	42.3	32.9	44.3	36.3	32.8	28.7	34.3	-
7	Esfahan	33.4	30.6	32.2	25.6	21.9	23.3	31.4	37.6	33.1	31.8	38.9	41.9	50.3	32.5	27.6
8	Isfahan	31.4	28.5	30.1	26.5	22.9	26.2	25.9	28.8	27.2	26.8	33.2	39.1	59.0	26.4	23.8
9	Shadgan	30.3	25.6	30.1	25.9	20.7	27.3	25.0	36	23.2	39.1	41.2	35.7	34.4	32.8	-
10	Khoramshahr	30.3	37.6	29.7	20.8	19.9	23.4	25.5	25.8	24.9	29.8	31.5	38.3	57.0	-	-
11	Tehran	29.0	30.5	24.5	19.7	16.3	21.4	26.2	27.5	26.8	32.2	38.7	34.8	49.1	25.9	26.1
12	Abyek	27.3	53.0	35.4	67.8	50.9	17.9	19.6	16.4	15.1	20.2	25.8	14.0	19.0	-	-
13	Dezful	27.1	26.4	21.7	19.0	21.8	33.6	23.9	30.1	24.9	36.9	33.1	29.8	22.6	26	-
14	Shahin Shahr	27.0	30.2	29.9	25.7	26.0	26.2	22.9	24.9	23.8	25.1	23.2	28.3	36.5	26.1	-
15	Karaj	26.8	29.7	24.3	24.2	18.9	22.9	22.4	20.1	18.3	27.7	30.0	33.8	48.3	23.0	22.2
16	Shiraz	26.8	20.4	21.2	20.3	23.9	24.2	27.1	24.0	26.5	26.2	34.7	39.8	39.9	25.1	-
17	Nazarabad	26.1	-	-	18.6	18.5	20.8	28.4	26.9	23.5	25.4	30.5	30.3	39.3	24.8	-
18	Mashhad	25.6	30.3	26.7	21.7	15.2	18.0	25.3	24.1	22.0	26.0	27.8	32.5	37.4	25.1	30.6
19	Malard	24.7	23.8	22.3	14.5	15.8	24	20.6	24.1	15.9	19.2	39.3	33.0	41.0	-	-
20	Nehbandan	23.9	14.5	62.3	39.4	52.0	30.0	23.0	27.3	18.6	13.8	15.4	10.1	8.0	-	-
21	Khoramabad	23.5	19.6	16.4	15.3	29.1	37.2	29.8	32.7	30.3	27.4	19.1	15.3	11.0	-	-
22	Najafabad	23.3	20.5	18.3	14.8	15.3	18.1	24.3	25.0	28.4	27.3	27.9	29.0	41.5	20.6	-
23	Mobarakeh	22.5	21.0	20.6	18.7	18.7	18.2	15.2	18.5	24.5	21.7	27.8	28.5	34.4	21.3	24.1
24	Qasr-e Shirin	21.8	22.1	25.2	25.6	17.4	22.7	17.4	18.1	14.7	19.1	24.5	30.5	23.7	-	-
25	Fahraj	21.6	17.2	33.0	21.4	27.9	17.6	23.2	28.9	23	18.9	16.8	19.1	18.3	-	-
26	Qorveh	21.4	13.2	12.8	-	15.2	26.5	21.6	25.0	24.5	29.5	27.3	18.0	16.8	13.7	7.8
27	Pardis	21.4	20.4	18.6	14.7	14.8	22.8	29.1	22.2	20.6	21.9	25.8	18.7	27	16.9	-
28	Saveh	21.3	20.1	17.4	16.0	16	22.8	22.9	24.6	21.6	22.5	22.3	23.9	28.2	19.8	-
29	Lavasan	20.8	25.8	21.5	16.5	12.4	17.2	14.6	14.4	14.6	22.1	26.2	22.2	37.4	-	-
30	Qom	20.3	15.1	16.1	8.4	11.2	15.3	21.6	24.0	18.5	18.8	21.6	23.0	38.8	-	-
31	Kerman	19.8	15.1	25.0	25.7	26.3	17.3	19.9	16.7	21.8	15.0	18.2	22.6	24.5	19.9	24.4
32	Meybod	19.2	23.7	11.9	12.3	-	10.9	11.5	-	18.5	19.1	18.0	24.2	32.5	22.0	21.1
33	Hamedan	18.8	26.3	19.7	13.5	14.2	29.3	14.6	18.0	16.1	21.1	20.8	18.3	22.5	21.8	-
34	Yazd	18.5	12.6	17.9	12.9	17.0	16.4	20.3	20.6	23.9	18.3	17.6	21.5	24.0	13.7	-
35	Arak	18.2	18.2	18.5	14.2	12.1	16.3	9.4	15.8	17.3	23.1	27.2	18.8	26.8	21.7	-
36	Tabriz	17.6	26.1	19.6	12.8	14.6	14.2	13.0	13.6	10.2	17.0	21.3	20.4	28.5	13.2	12.2
37	Semnan	10.6	-	13.6	10.3	12.4	11.5	8.5	5.4	10.5	10.9	10.3	9.7	11.6	-	-
38	Sanandaj	9.6	7.3	8.1	6.9	6.7	9.1	9.9	10.0	6.3	18.9	24.7	13.0	11.4	6.5	

Ahvaz is one of the world’s most polluted cities as a result of microdots blowing in from Iraq. The city’s particulate matter concentration is about three times that of Beijing (China’s capital) and 13 times that of London (Ghorani-Azam et al., 2016; Mawer, 2014). Major industries such as the steel and oil industry and drilling and oil extraction activities contribute significantly to Ahvaz’s air pollution. In addition to industry (Velayatzadeh, 2020), it was reported that In the winter months (November through February), the particular matter concentration was higher than in the spring and summer months (March through October). (Goudarzi et al., 2021). Similarly, air pollution in most cities also varied seasonally. It deteriorated after October, and pollution reached its peak in the winter. Atmospheric temperature inversion worsens air pollution during that period. Nevertheless, the end-of-year numbers go beyond what was normally acceptable, reaching 43.9 g/m^3^ (unhealthy) for sensitive groups. Shooshtar, the city located 92 km to the northeast of Ahwaz, with a PM2.5 monthly concentration of 55.9 μg/m^3^ in November, had the highest concentration in 2019 in Iran. The city of Varamin, located 60 km south of Tehran, recorded an annual average of 33 μg/m^3^, placing it at the third rank among all Iranian cities. Once again, November and December were the most polluted months, by quite a bit. It was in April that Varamin recorded its cleanest month (23.7 g/m^3^). Contrary to this, November and December recorded PM2.5 readings of 48.8 μg/m^3^ and 50.6 μg/m^3^ respectively (IQAir, 2021). Concerning Iran’s capital city, Tehran has more than 17 million vehicle trips daily many of which have outdated technology. Topography and climate are adverse to the pollution issue since it is at a high altitude and is surrounded by ranges of mountains (Alborz Mountain), which trap polluted air.

Moreover, the temperature inversion phenomenon occurs especially in winter, which can prevent pollutants from being diluted (Martin Heger & Sarraf, 2018; Hosseini & Shahbazi, 2016; Shahbazi et al., 2016, 2019a, 2019b). With regard to Tehran Air Quality Control Company’s annual reports (Air Quality Control Company 2021), within the Persian year of 1399 based on the solar calendar (2020–2021), Iran had 17 clean days (5%), 226 acceptable days (62%), 107 unhealthy days for sensitive groups of society (29%), and 16 unhealthy days (4%); compared to the previous year, 22 days (6%) has been added to the number of undesirable days. Analyses of carbon monoxide (CO) concentration over the studied period reveals that Tehran’s air quality due to CO was healthy. Although the reduction in the sulfur content of fuel distributed in Tehran in recent years has led to a decrease in the concentration of sulfur dioxide, the records of the studied year indicate a slight increase in SO_2_ levels from the previous year.

**Fig. 5:**
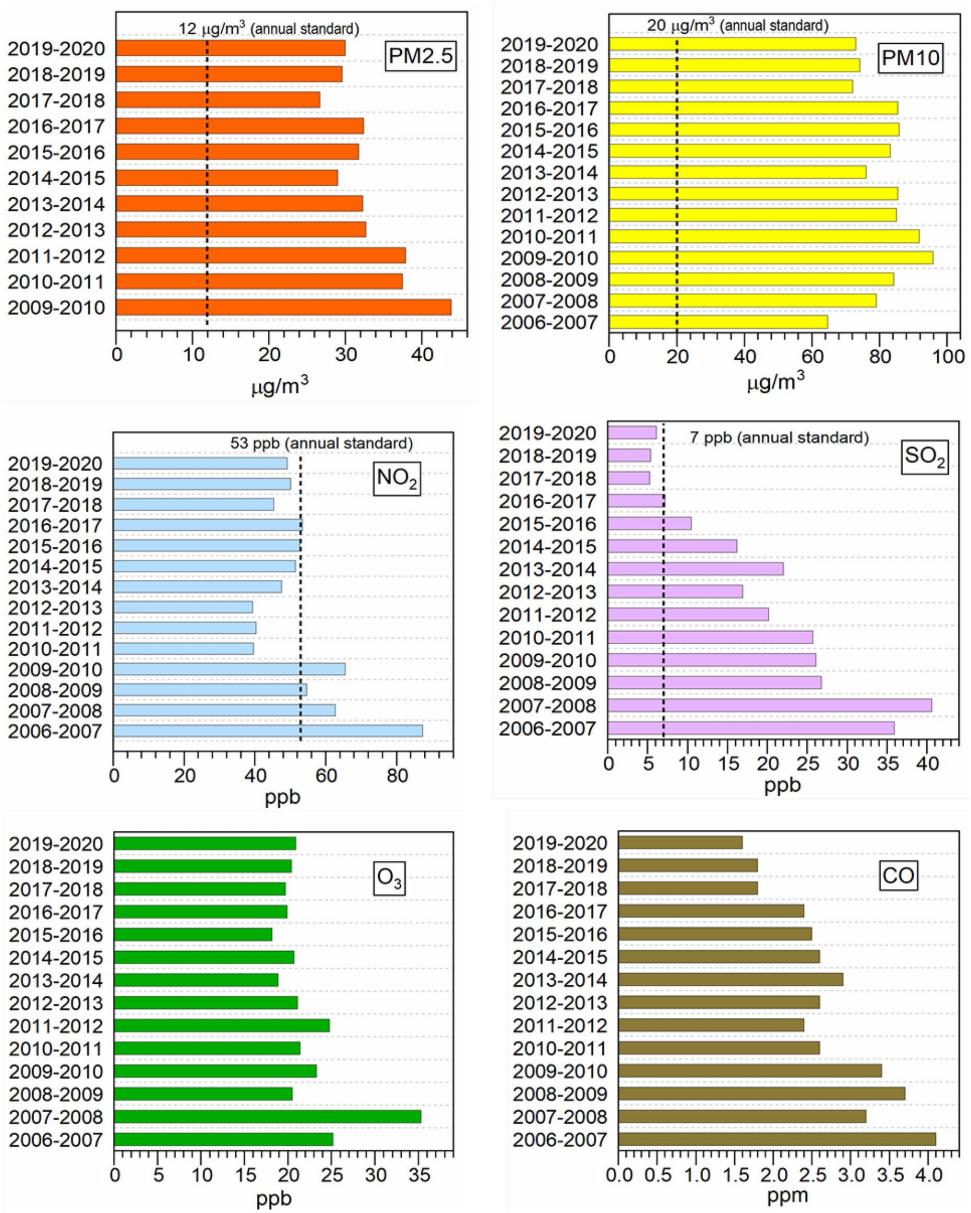
Main pollutant concentrations in Tehran (capital city of Iran), based on data reported by (Air Quality Control Company 2021)

Finally, the ozone levels remained in healthy conditions during most days of this period, except for 42 days with elevated levels in the summer. According to the daily national standard levels, the level of nitrogen dioxide (NO_2_) concentrations remained below the standard limit for health conditions on most days during the mentioned period and only 5 days were beyond the daily standard limits. However, considering the annual average national standard levels of NO_2_, recorded in some stations, there were 6 days in the year 1399 (2020) in which PM10 concentrations measured were beyond the daily standard limits. Overall, the fine particulate matter (PM2.5) was considered to reflect unhealthy conditions in Tehran, and most of the polluted days were associated with an increase in this pollutant. As illustrated in Fig. 5, Tehran’s air quality monitoring stations measured particulate matter levels above the national standard (Banirazi Motlagh et al., 2021).

### Major sources of air pollution

Air pollution has several different emission sources. However, the majority of environmental pollutants are issued through large-scale human activities such as industrial processes, power generation, and vehicles (Ghorani-Azam et al., 2016). Fossil fuel combustion from mobile sources results in a large number of emissions. Depending on the location, mobile source emissions may contribute differently. This is dependent on the technical features of the fleet, the fuel quality, and the intensity of economic activities. Moreover, a recent study investigated heavy metal concentrations (Fe, Zn, Cu, and Ni) mainly result from on-road pollution (Bisht et al., 2022).

Industrial sections like manufacturing plants, refineries, and power stations as stationary sources contribute significantly to the emission of sulfur dioxide, particulate matter, carbon dioxide, and nitrogen oxides (Molina, 2021). Iran’s power plant sector contributes 22% of the country’s energy consumption and % of its carbon dioxide emissions (Danaci et al., 2020). Furthermore, Iran’s energy has been influenced by the growth in demand for energy-intensive products such as automobiles (Yazdan et al., 2012).

As cities grow, electricity demand has increased, leading to the development of power stations and their intense use of fossil fuels, resulting in a highly energy-intensive and polluting industry sector. It has been widely argued that because the government subsidizes the price of energy, Iran has become one of the world’s most energy-intensive countries; however, the country has also faced environmental concerns because of its growing and unregulated polluting industries.

Iran’s high energy intensity is caused by a number of factors, including rapid urbanization and industrialization, little interest in increasing industrial productivity, a lack of access to advanced technologies caused by factors such as sanctions, insufficient attention paid to research and development, and energy policies (Mazandarani et al., 2011; Hazrati & Malakoutikhah, 2019; Rajabi Kouyakhi and Shavvalpour, 2021). On the other hand, worldwide attempts are ongoing to transition from fossil fuels to renewables and to essentially alter the present global system, which is dominated by fossil fuels. A shift to renewable energy would have at least two significant advantages: first, it would reduce greenhouse gas (GHG) emissions, and second, it would increase energy security. Generally, Iran’s share of renewables in electricity production experiences an energy trend that has been downward.

The world’s percentage of producing electricity production has been rising over the past decade. It should be emphasized that Iran’s geographical location is suitable for various kinds of renewable energy, particularly wind and solar power, due to having high levels of solar radiation and 300 sunny days per year on average, as well as its capacity to generate 1.4 gig watts (GW) of wind power. Note that the average annual wind speed on Iran’s 2.1 million hectares of land is 8 m per second (m/s). However, due to low-cost fossil fuel resources and insufficient infrastructure, investment, and technology, the contribution of renewable energy in generating electricity was less than 6.9% in 2020 (2.9% less than in 2019) (Azadi, 2017; Hazrati & Malakoutikhah, 2019).

Transportation sector emissions are considered to be the most significant source of air pollution in large cities around the world. Vehicle emissions contribute to about a quarter of global energy–related GHG emissions and cause significant air pollution, mostly in residential areas. As a result of exhaust gases, the transportation sector contributes 70 percent of all air pollution (Sofia et al., 2020; Xia et al., 2015). Carbon monoxide, nitrogen oxide, volatile organic compounds (VOC), and sulfur oxide are primarily emitted by automobiles. As a result, motorcycles emitted more than 15%, 31%, and 12% of the total traffic emissions in carbon monoxide, VOCs, and particulate matter, respectively. The nitrogen oxides, sulfur oxides, and particulate matter emissions from medium- and heavy-duty vehicles accounted for more than 41%, 64%, and 85% of Tehran’s fleet vehicles (Shahbazi et al., 2016). Due to Iran’s abundance of natural gas resources, natural gas use in the automotive industry seems logical. It is one of the pioneer countries replacing retrofitted CNG vehicles with gasoline-powered ones due to massive investments in replacing petroleum with natural gas as the standard fuel for its transportation fleet. However, it is essential to study the associated problems and factors of these types of vehicles. Apart from the benefits of using natural gas, the outdated dual-fuel technology, outdated conversion kits, and lack of regular inspections make it difficult to determine whether converted vehicles will contribute to reducing air pollution (Hashemian et al., 2013).

### Air quality management process

**Fig. 6:**
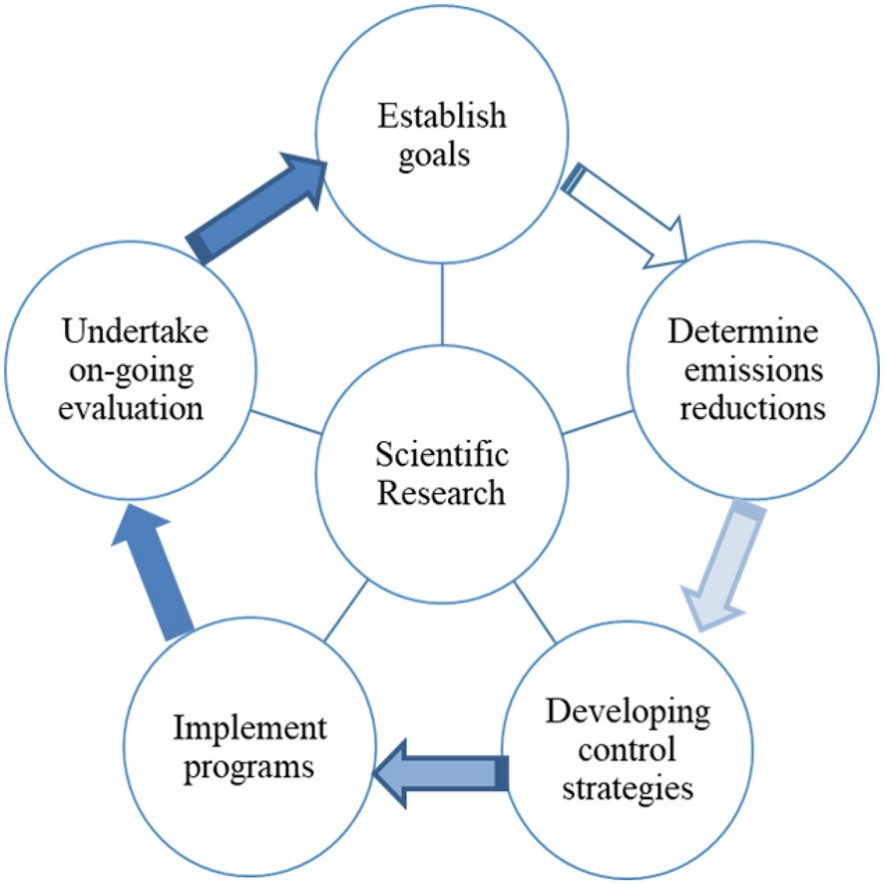
Air quality management process (based on the EPA (2021) definition)

Revisiting the air quality management process might be helpful to gain insight into current weaknesses by considering the possible solutions. The term “air quality management” refers to the systematic monitoring and evaluation of strategies aimed at reducing air pollution levels sufficiently in urban areas. Efficient pollution control measures rely on a thorough understanding of their costs, emission inventories, and clear goals for reducing emissions (Gulia et al., 2015; Vlachokostas et al., 2009). According to EPA (2021), the air quality management process can be illustrated with a cycle of linked factors. This process is presented in Fig. 6.

The starting point of air quality management is setting broad (protect human health and the environment from the adverse effects of air pollution) and specific goals such as research on different aspects of air pollution. In the second step, desired emission reduction should be determined by experts to achieve the goal by applying adequate tools such as emissions inventories, pollutant monitoring, and air pollution modeling. In developing control strategies as the third phase, air quality managers consider strategies in order to succeed in reaching the desired goals. In the fourth stage, implementation programs are essential for the effective control of pollution. It is necessary to enact legislation that will result in the reduction of emissions (Gulia et al., 2015). Finally, keeping track of air quality goals requires regular inspections and strategies based on their effectiveness. In this term, the role of scientific research that provides essential information is not deniable. It is worth mentioning that enhancing the continual improvement in air quality, legislation, and related goals should be updated and revised regularly based on state-of-the-art research and technologies. Besides, experts should provide constant feedback on progress and programs.

Since 1987, the WHO has issued a guideline to minimize the effects of air pollution on public health. The air quality standards of each country are different in terms of their policy, available technology, economic, social, and political factors, and the ability of each country to manage air quality. In 2020, aiming to provide more specific data, the United Nations Environment Programme (UNEP), in partnership with the UN-Habitat and IQAir (EPA, 2021) developed the largest real-time air quality databank, compiling real-time data on air pollution from various sources. As part of this collaboration, in more than 7000 cities around the world, governing agencies can improve air quality while empowering citizens to make smart decisions about their health and lifestyle (Molina, 2021).

**Fig. 7:**
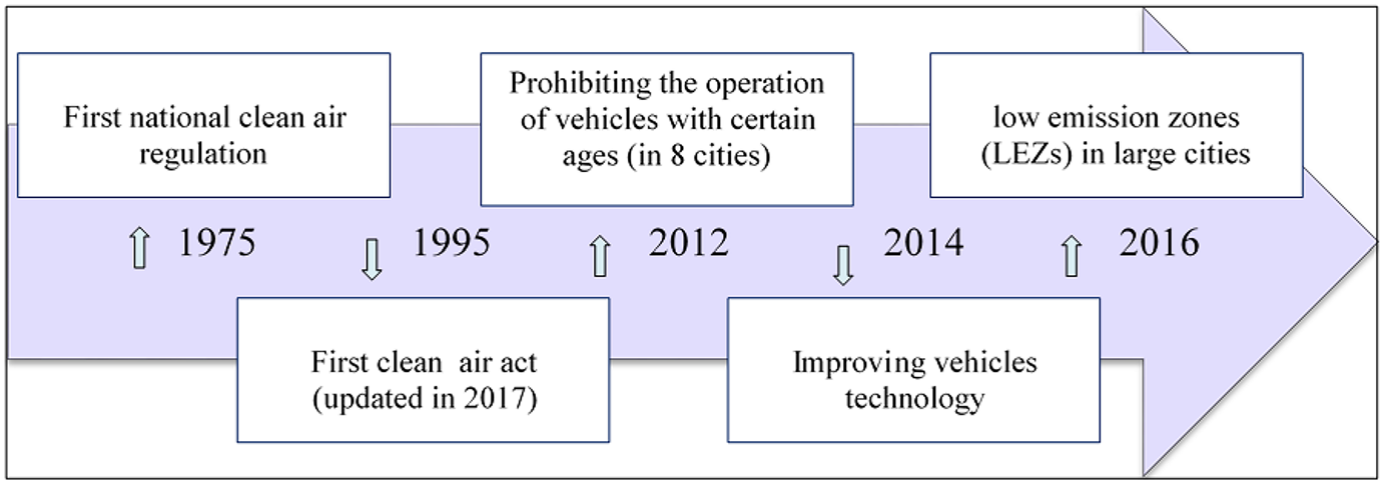
Timeline of air pollution regulation in Iran

In this case, Iran made a strong commitment to air quality monitoring in terms of regulations, policies, technologies, and fiscal at both the broad transport level and the vehicle level. Figure 7 illustrates a timeline of national legislation since 1975. According to Clean Air Act 1995, the Department of Environment (the DoE) is responsible for controlling. This legislation consists of six chapters and 36 sections and classifies the air pollution sources into three groups: motor vehicles, factories, workshops and power plants, business, and domestic and miscellaneous sources (Asadollah-Fardi, 2003). Although laws, regulations, and standards are in place, implementation, oversight, and enforcement are not as effective as they could be.

It was evaluated that regulations are not enforced effectively by the environmental protection organization because it has limited authority (UNEP, 2015). Asadollah-Fardi (2003) points out that ineffective enforcement may be caused by a lack of a management system that forces the authorities to adhere to the program, due to a lack of funds, and equipment expertise. In terms of public transportation, over the past decades, transport has substantially increased which leads to decelerate emissions even in the face of population growth and urbanization. Nevertheless, although it is obvious that a great deal of effort and control action has been undertaken, the desired outcome was not achieved. The final revision (2019) of the ambient air quality standards of Iran (Air Quality Control Company 2021), as well as WHO guidelines, is presented in Table 3.

**Table 3:** Ambient air quality standards of Iran and WHO guidelines

Pollutant	Iran standards (based on EPA)		WHO guidelines	
	Averaging time	Level	Averaging time	Level
PM2.5	24 h	35 μg/m^3^	24 h	25 μg/m^3^
	Annual	12 μg/m^3^	Annual	10 μg/m^3^
PM 10	24 h	150 μg/m^3^	24 h	50 μg/m^3^
			Annual	20 μg/m^3^
O_3_	8 h	0.07 ppm	8 h	100 μg/m^3^
NO_2_	Annual	53 ppb	Annual	40 μg/m^3^
	1 h	100 ppb	1 h	200 μg/m^3^
SO_2_	1 h	75 ppb	10 min	500 μg/m^3^
	24 h		24 h	20 μg/m^3^
Pb	1 month	0.15 μg/m^3^	Annual	0.5 µg/m^3^
CO	8 h	9 ppm	-	-
	1 h	35 ppm		

It is possible to involve citizens in developing measures to improve air quality by providing them with information about air quality named Air Quality Index (AQI). That is including PM2.5, PM10, nitrogen dioxide, sulfur dioxide, and ozone ranging from 0 to 500 as indicated in Table 4.

**Table 4:** Color concept and value range in Iran Air Quality Index (source: https://aqms.doe.ir/)

Color	AQI	Health indicator	Description
Green	0–50	Good	Air quality is considered satisfactory, and air pollution poses little or no risk
Yellow	51–100	Moderate	Air quality is acceptable; however, for some pollutants, there may be a moderate health concern for a very small number of people who are unusually sensitive to air pollution
Orange	101–150	Unhealthy for sensitive groups	Members of sensitive groups may experience health effects. The general public is not likely to be affected
Red	151–200	Unhealthy	Everyone may begin to experience health effects; members of sensitive groups may experience more serious health effects
Purple	201–300	Very unhealthy	Health alert: everyone may experience more serious health effects
Maroon	301–500	Hazardous	Health warnings of emergency conditions. The entire population is more likely to be affected

Nowadays, most countries have implemented air quality management systems. However, these measures are strongly influenced by local economic conditions and technological availability (Sofia et al., 2020). Nevertheless, achieving the goal of air quality requires effective policies and strategies to be implemented at a sufficient scale. In this regard, in the first place, assessment of the current state of air quality management on a regular basis seems essential. Schwela et al. (2012) suggested that this assessment can be done in four criteria including air quality measurement capacity, data assessment and availability, emission estimates, and management enabling capabilities. Furthermore, they argued that in most Asian cities, the main weak point in air quality monitoring is related to quality assurance and control procedures. A strict quality assurance plan is often not developed or implemented. Therefore, such air quality data cannot provide the basis for a detailed evaluation of air quality trends in air quality, source apportionment, compliance with standards, meeting compliance with standards, and environmental and environmental impact. As a consequence, policy making and enforcement of emission regulations may be weakened.

To begin with, emissions must be analyzed regionally and periodically to identify their contribution from different sources, but unfortunately, such an inventory is not available. Further improving emission inventories and developing better projections will enhance the capability of monitoring and modeling to facilitate the development of policies (Gulia et al., 2015).

Finally, regarding the importance of monitoring and data reporting systems, it could be said that knowledge comes from measurements, particularly in the air quality context, our evaluation strongly depends on air quality measured data. In this regard, to develop cognition and knowledge through this global issue, more focus should be taken on: establishing an air pollutant measurement system, applying critical evaluation, and data methods, and enforcing local authorities to publish regular (monthly and annually) reports.

### Literature review on the “Air pollution in Iran” field

The ambient air pollution in Iran has become one of the most concerning and challenging environmental issues for local authorities as well as national and international researchers establishing the necessity of appropriate sustainable control policies and regulations against all aspects of various issues of air problems. Thus far, several pieces of literature have been focused on the issues of Iran’s air pollution including the investigation of the nature and source of air pollutants and their adverse health effects, and emission inventory. Several of these studies are listed in Table 5.

**Table 5:** The previous literature review on the subject of air quality in Iran

Location	Year	Main results	Ref
Tehran	2023	Presenting a platform for air pollution detection, estimation, and control in cities like Tehran	(Kiyan et al., 2023)
Zahedan	2023	The impact of PM10 on the health of citizens in Zahedan	(Leili et al., 2023)
Tabriz	2022	An examination of long-term changes in pollutant levels	(Barzeghar et al., 2022)
Tehran	2022	Effect of PM concentration relative humidity and wind speed on SO_2_ trend in Tehran machine learning analysis	(Borhani et al., 2022)
Iran	2022	Exposure to COVID-19 was positively correlated with PM2.5 levels	(Juarez et al., 2022)
Tehran	2021	CO_2_ and PM10 are more affected by the replacement of motorcycles, while NOx and SOx are more affected by replacing cars with a bicycle	(Monazzam et al., 2021)
Tehran	2020	Most and least levels of all air pollutants were observed during the summer and winter, except for O_3_	(Yousefian et al., 2020)
Isfahan	2020	Study the social aspects of air pollution	(Jokar et al., 2020)
Iran worldwide	2019	From 1990 to 2016, ambient particulate matter levels in Iran exceeded WHO guidelines	(Shamsipour et al., 2019)
Tehran	2019	Using emission inventories to assess the impacts of Tehran’s pollution policies	(Shahbazi et al., 2019a, 2019b)
Tehran	2019	PM is the major cause of Tehran’s air quality decline, and in the cold seasons of the year, air quality in most regions of Tehran is in unhealthy conditions,	(Farhad Taghizadeh et al., 2019)
Tehran	2019	By developing an emission inventory, the relative contribution of major air pollutants from different mobile sources was identified	(Shahbazi et al., 2019a, 2019b)
Ahvaz	2019	In the cold and warm seasons, the average ambient PM concentration from both internal and external sources was 158 and 161 µg/m^3^, respectively	(Goudarzi et al., 2021)
Tehran	2019	If passenger cars and motorbikes were removed, it could significantly reduce PM2.5 and NO_2_	(Shahbazi et al., 2019a, 2019b)
Tehran	2018	The lasting effects of ambient PM2.5 and O_3_ on death rates have been studied in Tehran	(Karimi & Shokrinezhad, 2021)
Tehran	2018	An overview of the severity of air pollution in Tehran	(Martin Heger & Sarraf, 2018)
Ahvaz	2018	Confirming negative short-term effects of air pollution on death from cardiovascular disease in Ahvaz. Emphasizes the importance of implementing policies to reduce air pollution	(Dastoorpoor et al., 2019)
Iran worldwide	2016	In spite of national air pollution programs, there is not much progress, coordination is weak, and funding sources are limited	(Hosseini & Shahbazi, 2016)
Isfahan	2015	SO_2_ concentrations are heavily influenced by industries and power plants, long-term wind patterns, meteorological factors, and temperature	(M Heger & Sarraf, 2018)
Tehran	2001	Regulations are in place, but implementation is not satisfying may be due to the presence of some weakness	(G. Asadollah-Fardi, 2003)

## Potential mitigation solutions

Implementing a long-term strategy based on local economic and policy conditions is essential for providing significant benefits simultaneously to the environment and health (Sofia et al., 2020). Modern and environmentally friendly technologies and effective regulations can reduce air pollution emissions significantly. In practice, there are a number of obstacles, such as the country’s economic status. Effective and realistic strategies and comprehensive emissions reduction measures need to be identified and implemented at an adequate scale (Bui et al., 2018; Schwela et al., 2012). A number of common reduction strategies are highlighted in the previous literature such as the development of traffic management systems, the development of public transportation and infrastructure, the adoption of electric vehicles, the introduction of modern policies, and the establishment of systems for monitoring air quality that are more advanced (Anjum et al., 2021; Gulia et al., 2015; Parrish et al., 2016; Schwela et al., 2012). Herein, with regard to the current situation in Iran which has been highlighted previously, some essential strategies and considerations are suggested that can be used not only in Iran but also in other developing countries.

### Scientific research

As mentioned before, scientific research is a key element in air quality management. Systematic studies can provide essential information such as:

- Provide emission inventory and consider the up-to-date status of air pollution regularly.
- Provide environmental and engineering studies on air pollution control strategies and technologies.
- Investigate the effectiveness of policies and strategies to reduce air pollution.
- Explore the impact of previous policies, regulations, and strategies.
- Highlight potential co-benefits of air pollution strategies concerning GHG emissions.
- Promotion of renewable alternative fuels and new innovative technologies.

Despite, as mentioned previously, the numerous publications conducted on air pollution in Iran, most of these investigations are still scattered and a systematic review remains lacking. Recently, several scientometric reviews as a reliable and valuable method for identifying patterns in scientific research and obtaining scientific findings have been conducted to show an accurate picture and trend in pollution and its association fields (Hamidi & Ramavandi, 2020; Kumar et al., 2023; Kumar et al., 2023; Ranjbari et al., 2023). Using such methods, outputs would highlight the need to focus on the study area and provide insight into the issue. Performing such research on air pollution topics in Iran, such as health issues, monitoring technology, and reduction technologies, would provide a better understanding of the trend in organized research as well as the associated costs and reduce the amount of research repetition required by authorities and respected institutions.

### Public awareness

Citizens should have the legal right to free access to air quality information and public participation to freely obtain it. As a result, residents have the right to request details about national air quality plans. Air quality information should be available promptly and appropriately, particularly when air quality standards are surpassed by national government agencies. Moreover, any social acceptability primarily needs individuals to understand the complexity and benefits of new technologies, legal interventions, or behavioral changes. In this context, programs such as increasing the attractiveness of public transport, use of better-quality fuels, and educating the public about the importance of pollution control measures, eco-friendly activities, and transportation can be considered mitigation strategies (Grossberndt et al., 2020; Maione et al. 2021). Nevertheless, public awareness, particularly in developing countries, can lead to a change in citizens’ behavioral patterns (Nahar et al., 2021). In the case of Iran, although many efforts have been made to raise people’s awareness of the issue in various media, it should be noted that this awareness will be effective as long as citizens receive adequate training and practices regarding the ways in which they can contribute to the reduction of air pollution.

### Implement an integrated approach

This approach takes into account the co-benefits of integrating climate change and air pollution. However, these two related environmental issues remain independent and are addressed by different scientific communities under distinct policy frameworks. There are lots of mitigation strategies beneficial to one side, but exacerbated by the other. For instance, a purely environmental approach to reducing carbon dioxide emissions by encouraging the use of wood-burning stoves, diesel, or biofuels can negatively impact air quality. So, a coordinated effort is needed, taking into account the link between air quality and climate (Maione et al., 2016). Practical strategies are suggested below:

1. Identifying and formulating policies that integrate these two environmental issues in order to achieve co-benefits.
2. Promoting and encouraging the production and consumption of cleaner energy.
3. Enhancing air quality while reducing carbon dioxide emissions through the use of renewable energy sources.
4. Establishing and developing partnerships with international organizations to share knowledge and resources on air pollution and climate change.

### International collaboration

In order to measure emissions, track air pollution and its impacts, and identify cost-effective measures, international policy collaboration on air pollution science is crucial. Air quality improvement in different areas around the world can provide valuable perspectives for other countries facing this challenge. An investigation of integrated air quality management in Mexico City and Singapore by (Molina et al. 2019) as well as an analysis of air quality improvement in Los Angeles (Parrish et al. 2016) provide examples of this kind of research. Unfortunately, there are severe international sanctions on Iran; this has caused barriers and resulted in difficulties for researchers in the field to freely collaborate with their peers worldwide. Examples can be the use of industry-standard software and purchasing measurement devices to use for R &D in the laboratories were of concern for some time. In this regard, prioritize collaborations with international peers to share knowledge, resources, and expertise.

## Conclusion

In recent years, air pollution has been one of the most prominent environmental concerns that have been faced by large cities throughout Iran. This not only causes adverse effects on individuals’ health and counts as the main reason for enormous premature death but also results in catastrophic effects on the environment and climate change. As air pollution issues become one of the most concerning issues in Iran, it is important to remain informed about the current status of air pollution and its association. In the current study, we sought to provide an overview of the current information and publications regarding “air pollution in Iran.” By overview, the current information and published report of this study establish that most Iranian cities have witnessed prolonged periods of pollution during the winter, dust phenomena, and an increase in ground-level ozone during the summer, with some pollutants exceeding national standard limits. For instance, PM2.5 concentrations reported at air quality monitoring stations surpassed the standards, indicating unhealthy conditions, and it is also worth mentioning that most polluted days were associated with an increase in the concentration of this pollutant. More recent data on the impact of COVID-19 was also addressed. It was found that during the national lockdown amount of particulate matter increased to about 10.68% due to the use of private cars rather than the use of public transport. Potential mitigation solutions to overcome the challenge of air pollution were also discussed. This is including but is not limited to public awareness, scientific research, and international collaboration. However, despite the planned activities carried out to reduce air pollution, air quality is still critical. Finally, regarding this paper, we suggest that further research should focus on providing systematic reviews using data mining or machine learning approaches to show the accurate picture research trend in this field. It would be beneficial for authorities and policymakers to consider addressing climate change and air pollution issues simultaneously, as well as collaborating with international counterparts to share knowledge, tools, and techniques.

## Data Availability

All data are available.

## Abbreviations

AQCC: Air Quality Control Company
AQI: Air Quality Index
BC: Black carbon
CH_4_: Methane
CO_2_: Carbon dioxide
CO: Carbon monoxide
CNG: Compressed natural gas
DoE: Department of Environment
EPA: Environmental Protection Agency
GHG: Greenhouse gas
GW: Gig watts
LEZs: Low emission zones
NO_2_: Nitrogen dioxide
NO_X_: Nitrogen oxides
Pb: Lead
PM: Particulate matter
O_3_: Ozone
SO_2_: Sulfur dioxide
UNEP: United Nations Environment Programme
VOC: Volatile organic compounds
WHO: World Health Organization
